# Geospatial suicide risk prediction in the State of Maryland and the limits of discernible intent

**DOI:** 10.21203/rs.3.rs-10832081/v1

**Published:** 2026-09-20

**Authors:** Christopher Kitchen, Anas Belouali, Ayah Zirikly, Paul S Nestadt, Holly C Wilcox, Hadi Kharrazi

**Affiliations:** Johns Hopkins University; Johns Hopkins School of Medicine; Johns Hopkins Whiting School of Engineering; Johns Hopkins School of Medicine; Johns Hopkins University; Johns Hopkins University

**Keywords:** suicide risk, social determinants, geospatial clustering, undetermined manner of death

## Abstract

**Background.:**

The rate of suicide death is known to be unequally distributed by geography and associated with multiple sociocultural and clinical risk factors. It is less clear whether the same patterns exist for deaths where the manner is undetermined, acknowledging that it is not always possible for a medical examiner to know whether death is intentional.

**Methods.:**

Cumulative counts of suicide and undetermined death were aggregated to the Census tract level of the State of Maryland, between 2012–2020 and used as primary outcomes of interest. We evaluated death rate using two statistical approaches: 1) negative binomial models and 2) SaTScan geospatial clustering of pooled cases.

**Results.:**

Model effects for suicide reveal a marginal association with older age, and greater area deprivation index (ADI), but an inverse association with proportion of minority race population. Undetermined death was found to be significantly associated with ADI. SaTScan clusters for suicide revealed a small number of large clusters covering rural areas of Maryland, but many small clusters around metropolitan areas for undetermined manner of death.

**Conclusions.:**

The two techniques identified distinct regions of interest for public health surveillance and intervention planning, and with respect to each manner of death. Suicide deaths were found to be elevated in non-metropolitan areas. Limited access to care and increased social burden are found to be associated with undetermined death. There is more uncertainty around intent for these cases, often due to a lack of diagnoses, treatment or other clinical context.

## BACKGROUND

Suicide is consistently among the leading causes of mortality in the United States. The national rate of suicide death has increased steadily for more than 15 years, reaching a pre-COVID pandemic peak of 14.2 deaths per 100,000 in 2018, and 14.4 deaths per 100,000 more recently in 2022. [1–2] Suicide rate trends have varied greatly among individual communities and demographics, however. [3–4] Adolescents and racial or ethnic minorities are among those that have continued to see increasing trends over time, especially throughout the COVID-19 pandemic. [4] The full impact of these disparities is difficult to assess fairly, however, with some research suggesting there is a systemic pattern of misidentifying suicides, especially among increasing numbers of overdose deaths where intent is uncertain to medical examiners. [5, 6] It seems at least plausible that socio-cultural factors, unevenly distributed across geography, are partly to blame. [7, 8]

The United States is culturally and geographically diverse, so it is essential that public health researchers understand which factors influence the rate of suicide for different communities, at an ecological scale. Recent historical events are also believed to have contributed significantly to suicide death in recent years, such as the ongoing opioid crisis, rise in gun ownership and the COVID-19 pandemic. [4, 8–14] It is also known that the rate of suicide is significantly associated with poverty, lack of access to care and income inequity. [15–20]

There are therefore many overlapping and related influences on mental health outcomes, like suicide. These may be spatially and temporally bound, with exacerbations from historical events, but also affected by policy or intervention. Suicide may be prevented by identifying ‘where’ and ‘when’ these changes occur at a population level, not just by contact with health settings. Prior research has established both generalizable and local effects of suicide risks at an ecological scale. [7, 19, 20] Much work is being performed at jurisdiction and zip code levels, but lower geographies could improve detection of nuanced or localized effects. [18, 21, 22]

Mental health advocates have also called for a multi-level approach, including community surveillance to address suicide. [7, 16] A large share of persons who died by suicide had no clinically discernable risk in the year prior to suicide. [23–25] Furthermore, it is difficult to reliably predict suicide risk using electronic records alone. Those at risk may be overlooked by busy primary care providers, have limited access to care, or not even report having mental health symptoms due to socio-cultural influences. [26–28] If providers were aware of these risks, and which segments of their community were likely to be experiencing them, they may be better equipped to properly assess at-risk individuals.

To this end, we use the Maryland Suicide Data Warehouse (MSDW) to evaluate rates of suicide and undetermined manner of death between 2012 and 2020. Our central aim is to quantify these rates in both contexts, using a variety of publicly available, ecological data. These include estimates for social determinants of health (SDH), access to care, and behavioral or mental health condition prevalence. As a secondary aim, we use a popular spatial scan statistic used by epidemiologists to cluster geographic regions of interest, so called “hot spots,” that are based on disease prevalence. [18, 22] Through simplifying these regions of interest into contiguous, determinate clusters, researchers may more clearly quantify associations between ecological factors and significant elevations of risk, aiding care providers and other stake holders in evaluating suicide risk for different communities, especially those in which it may be underreported due to limited ability for the medical examiner to discern intent.

## METHODS

### Participants and Setting

All data from the MSDW are linked by a deidentified patient identifier, assigned by the regional health information exchange, Chesapeake Regional Information System for our Patients (CRISP). Manner and cause of death are primary outcomes recorded by the State of Maryland’s Office of the Chief Medical Examiner (OCME) and linked to a set of geographic markers to reflect last known patient address, from the CRISP Registry.

We assessed 5,059 deaths by suicide between January 1st, 2012 and December 31st, 2020. After removing patients without a valid, Maryland Census tract and tracts containing fewer than 10 decedents, 4,824 (95.4%) decedents were identified for analysis. Aggregated counts were tabulated across 1,385 remaining Census tracts. Decedents where the manner of death is undetermined were aggregated the same way, with 10,616 (91.6%) of the original of 11,592 decedents remaining. All other manners of death were ignored for the purpose of this analysis.

Tract population, demographic composition and SDH were extracted from the 5-year estimates of the 2019 American Community Survey (ACS). [29] Additional ecological data were obtained from multiple recent publicly available sources: 1) the 2010 Rural-Urban Commuting Area codes (RUCA), published by the US Department of Agriculture, 2) medically underserved areas as defined by the Area Health Resource Files (AHRF) published by the Health Resources & Services Administration, 3) the 2021 PLACES survey, published by the US Centers for Disease Control and Prevention. [30–33] For each source of data, we selected the most recent annualized estimates prior to the onset of the COVID-19 pandemic in early 2020. The 2021 release of PLACES reflects model estimates of 29 measures, including behavioral and mental health conditions using data from the 2019 Behavioral Risk Factor Surveillance System (BRFSS). The 2010 RUCA designations were categorized based on the decennial Census and therefore not changed as of 2019.

### Variable Definitions

Several demographic factors were accounted for in our statistical models, including population, median age, percent that is male and percent that is a racial or ethnic minority, excluding those identified as Caucasian only. SDH are known to vary with disease prevalence and severity of illness for a variety of conditions, including those related to suicide risk. [7, 15–17, 19, 21, 34–35] The Area Deprivation Index (ADI) and its components cover a cross section of different types of SDH, including housing, vehicle access, income and economic opportunity. [14, 35, 36, 37] The ADI was calculated using a method detailed by Knighton et al. and served as the main SDH feature of our analysis. We converted raw scores to a national percentile rank where 1 is ‘least deprived’ and 100 is ‘most deprived’ to control for outliers of the raw score distribution.

Medically underserved areas (MUAs) are published by the HRSA and were selected for designation date on or before December 31st, 2019. We ignored MUAs identified as ‘withdrawn’ or ‘governor’s exemption’ within the current AHRF. A MUA is defined as a lack of primary care settings within each geography covered by the designation, the criteria for which is managed by the HRSA’s Bureau of Health Workforce.

Prior research has established a trend of comparatively higher suicide death rates for areas with low population density. [7, 38] We modified RUCA primary designations based on the 2010 Census to differentiate tracts because the State of Maryland has a limited number of genuinely non-metropolitan (rural) areas. Normally, there are 10 primary categories, ranging from (1) Metropolitan – core to (10) Rural. Levels were collapsed such that code 1 stayed the same, codes 2 & 3 reflect metropolitan – non-core, code 4 through 6 reflect micropolitan and 7 through 10 were now considered ‘small town’ or rural.

The 2021 PLACES estimates were used for behavioral and mental health condition prevalence, as a percentage of tract population. [32, 39] A workgroup of experts manually reviewed the 29 conditions covered by these estimates and identified 7 plausibly related to mental health and suicide related outcomes. These features include overall poor physical and mental health status, obesity, depressive and sleep disorders, alcohol misuse, and current cigarette smoking.

### Ecological Models

Tract level cumulative counts of suicide and undetermined deaths for the entire 2012–2020 period were used as the outcomes of interest. Multiple predictive features were already scaled as a rate (e.g., percent of residents), or otherwise converted to percent, percentile and ratio, not impacted by denominator size across tracts. All predictive models also include the 2019 Census estimated tract population to predict count of deaths.

Negative binomial regression was used to accommodate overdispersion for each model. Performance was evaluated using the pseudo R-squared statistic, analogous in this case to overall deviance explained. [40] Three models were evaluated at the tract level: 1) a base model consisting of area demographic features only, 2) the base model plus social determinants: the modified RUCA, national rank ADI, and MUA designation, and 3) the base model plus SDH and the selected prevalence estimates for conditions. Coefficients and 95% confidence intervals for each model were converted to incidence rate ratios (IRR) for deaths of either type to assist interpretation. Effects were considered significant if the 95% interval of IRR did not overlap a value of 1.

### Geospatial Clusters

The geospatial clustering of suicide cases in the MSDW was performed using the SaTScan algorithm to identify statistically meaningful clusters of deaths. This approach uses pooled counts of cases and area population to estimate the maximum likelihood ratio, testing for significant divergences from expected values. [41, 42] The SaTScan method considers cases across all adjacent regions of cluster centers in a circular search pattern, adding adjacencies until a stop condition is met (e.g., more than a maximum count of tracts, some proportion of total population is met, etc.). This approach has been widely used in communicable disease surveillance, and more recently to identify regions with elevated suicide rates. [43–45]

Clustering consisted of 999 Monte Carlo simulations allowing at most 50 pooled tracts for cluster size, to avoid solutions of too few, overly inclusive or large clusters. A cluster was considered significant for p-values less than 0.01. Cluster identification and choropleth mapping was conducted using the ‘rflexscan’ and ‘tmap’ packages of the R programming language. [46, 47] The resulting statistics, case counts, expected values and relative rate of death for clusters were then tabulated for interpretation. Clusters are identified in descending order of the log likelihood, meaning the first cluster labeled by the algorithm is the most significant but may or may not have the greatest relative risk. Geospatial heat maps can be inspected visually to understand patterns of dispersion more easily and compare the clustering approach to one based on a statistical model. As a supplemental analysis, and to further characterize and validate geographic trends by manner of death, we used the same clustering methodology for a small number of listed categories identifying causes of death per the OCME.

## RESULTS

Between 2012 and 2020, the State of Maryland experienced an annual suicide death rate of approximately 9.3 deaths per 100,000 residents. Manner of death was undetermined at a rate of approximately 21.4 per 100,000 (see Supplemental Table 1 for a detailed characterization of our sample). According to the 2019 Census estimates, Maryland had a population of 6,018,848, with a median age of 38.7 years, 48.5% being male, 41.7% identifying as a non-Caucasian racial or ethnic minority. Across 1,385 Census tracts in Maryland, 1,155 (83.0%) were considered metropolitan area- core, 228 (16.4%) were MUAs and the average nationally ranked percentile score for ADI was 36.2 (std dev = 27.9), in 2019. The average estimated prevalence of each condition as a percentage of tract population was 15.3% for alcohol misuse, 15.7% for cigarette smoking, 17.5% for depression, 13.7% for generally poor mental health, 33.4% for obesity, 11.2% for poor physical health, and 38.4% for reduced sleep.

The composition of each model was found to have improved pseudo R^2^ with the addition of variables for each step (Table 1). For suicide deaths, this improved from 0.379 to 0.417 (10%) with the addition of MUAs, RUCA categories and ADI, then 0.417 to 0.450 (7.9%) with the addition of PLACES estimates. Much of the baseline variance explained appears to result from the association of suicide death counts with total population, median age and percent minority race. For undetermined deaths, baseline deviance explained was much worse at 0.034, though significantly associated with population, percent male, median age and percent minority race. By adding ADI, RUCA and MUAs, the undetermined model improved from 0.034 to 0.436 (1,182.4%), putting it at par with deviance explained in the suicide models, then adding PLACES estimates improved it further from 0.436 to 0.605 (38.8%).

Multiple effects of the final model for suicide were found to be significant, including median age (IRR = 1.014), percent minority race (IRR = 0.992), MUA status (IRR = 0.840), national rank ADI (IRR = 1.005), small town or rural RUCA category (IRR = 0.810), prevalence of cigarette smoking (IRR = 1.087), prevalence of poor mental health (IRR = 0.938), prevalence of poor physical health (IRR = 0.953) and prevalence of reduced sleep (IRR = 0.983).

For undetermined deaths in the full model, many of the same effects were significant but a few were also considerably different in size or direction: percent male (IRR = 0.992), median age (IRR = 1.021), percent minority race (IRR = 1.003), MUA status (IRR = 1.137), national rank ADI (IRR = 1.008), small town or rural RUCA category (IRR = 0.409), prevalence of alcohol misuse (IRR = 1.060), cigarette smoking (IRR = 1.086), depression (IRR = 1.134), poor mental health (IRR = 0.906), obesity (IRR = 1.017), poor physical health (IRR = 1.032) and reduced sleep (IRR = 0.982). Undetermined deaths were more strongly and positively associated with reduced access to care, area deprivation, and higher rates of depression and alcohol misuse. Undetermined deaths were also found to be less associated with small town and rural areas than suicide deaths.

**Figure 1:**
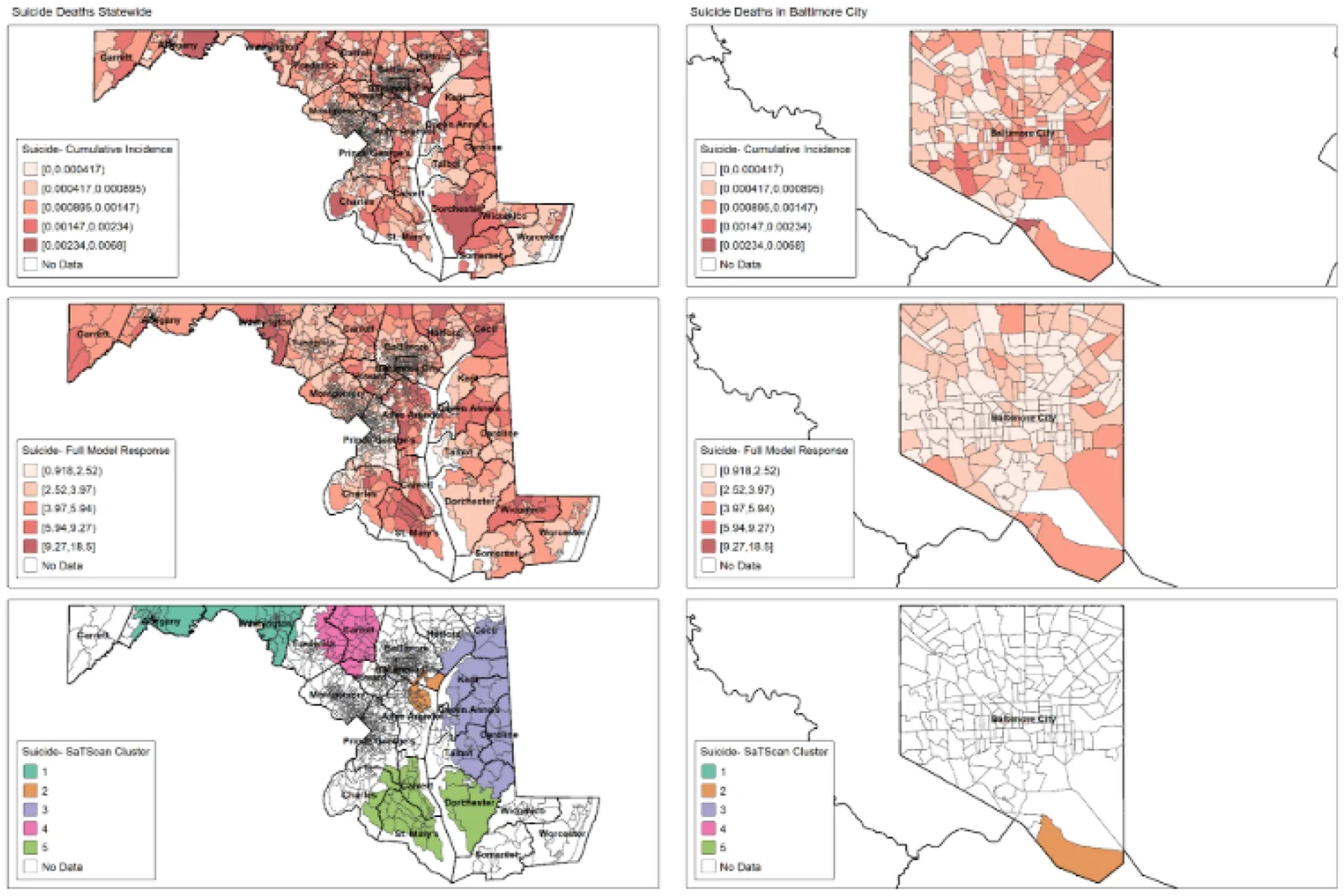
Gradient maps illustrating concentration of 1) cumulative suicide incidence 2012-2020 (top), 2) predicted count of suicide using model response (middle), and 3) SaTScan significant clusters for elevated suicide risk, for both the State of Maryland and Baltimore City.

**Figure 2:**
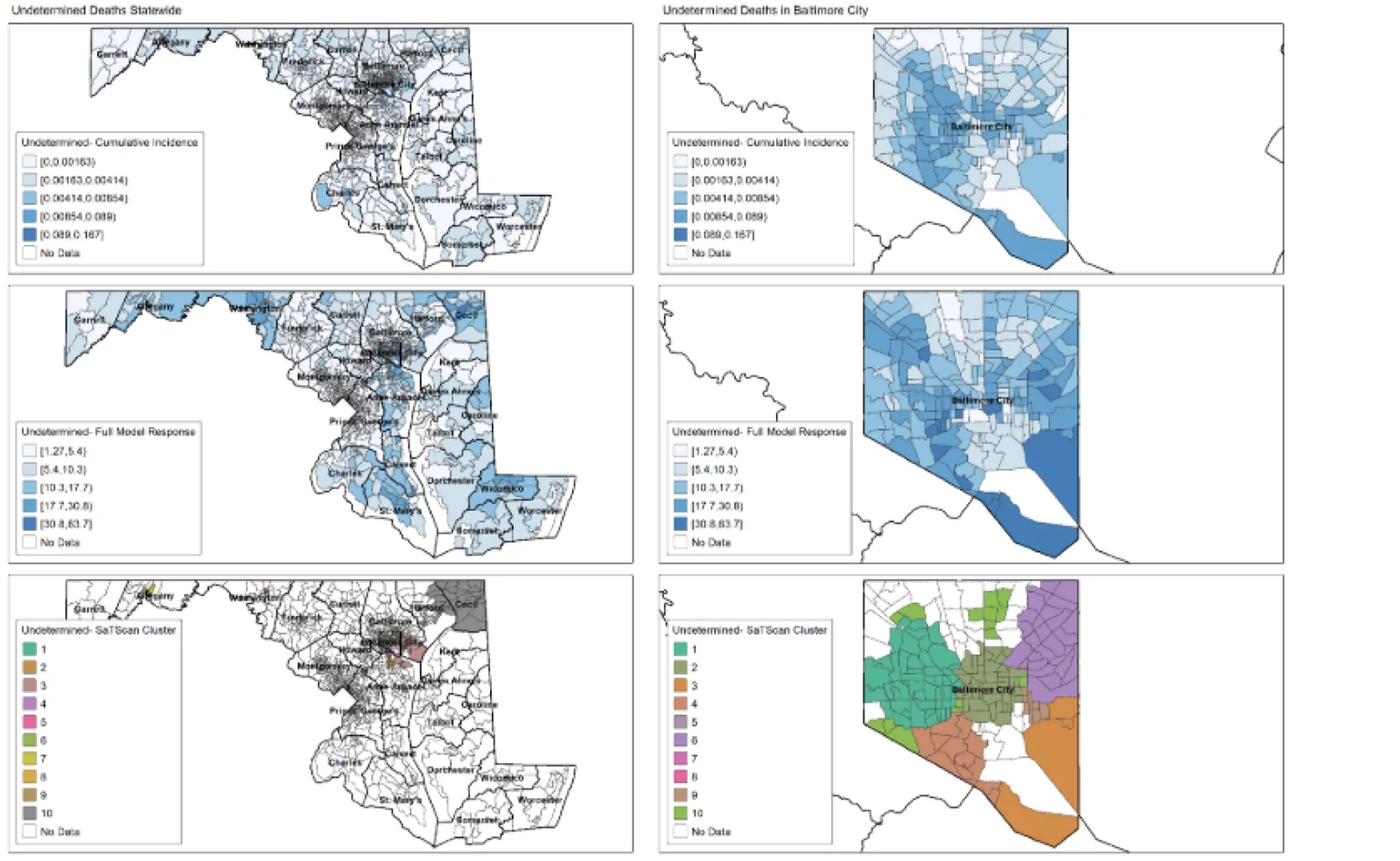
Gradient maps illustrating concentration of 1) cumulative incidence of undetermined deaths 2012-2020 (top), 2) predicted count of undetermined deaths using model response (middle), and 3) SaTScan significant clusters for elevated risk for undetermined death, for both the State of Maryland and Baltimore City. Clusters identified after the 10^th^ significant cluster are grouped with it to conserve levels for each display.

Figures 1 and 2 display the geospatial plotting of cumulative incidence of death (top panes), the predicted negative binomial response of full models (middle panes) and the SaTScan cluster assignments, which reflect significantly elevated counts for the suicide and undetermined manner of death (bottom panes). Death incidence and predicted model response are widely distributed across Maryland, but significant clusters are much more localized. Five statistically significant clusters were identified for elevated suicide rate, and eighteen substantially smaller clusters were found for undetermined manner of death (Supplemental Table 2). The SaTScan clusters for suicide reveal significant elevations for regions of western Maryland, Frederick and Carroll Counties, densely populated areas just to the south of Baltimore City, parts of southern Maryland and much of the eastern shore (Fig. 1).

Though the regions are large, we see that much of the region between Baltimore City and Washington DC is not part of these clusters, nor is it illuminated by the model. Figure 2 more clearly captures deaths in Baltimore City, only where it concerns greater than expected counts of undetermined deaths. Some regions outside of Baltimore also appear to have elevated rates of undetermined deaths but tended to be around population centers: just outside of Baltimore City, Cecil and Harford Counties and two cities in western Maryland.

SaTScan clustering of causes of death revealed many of same regions identified for suicide are those with elevated firearm deaths, while overdose or intoxication-related death appeared to be more consistent, geographically, with areas with greater undetermined deaths (Supplemental Figs. 1 & 2). This was confirmed by inspecting average estimated incidence of these causes by model response and cluster for both manners of death (Supplemental Table 3).

Finally, we assessed area characteristics for regions identified by either the predictive models or the SaTScan clusters for either outcome (Tables 2 & 3). For suicide, the regions associated with the highest elevations in model-estimated risk are characterized as less densely populated, have a smaller proportion of minority race, have fewer core metropolitan areas, less MUAs and area deprivation. For the highest risk cluster, population density was low, as was proportion minority race and MUAs, ADI was high and comparatively more tracts fell in metropolitan cores. Combined, the top decile of predicted risk accounts for 16.8% of suicides using 139 tracts and the five clusters accounted for 25.9% across 225 tracts. For Undetermined deaths, the top decile was found to be associated with increased population density, greater proportion minority race, more metropolitan cores, more MUAs and ADI, consistent with the first cluster found to be statistically significant. The top decile of predicted risk for undetermined deaths accounts for 20.5% of those observed with 139 tracts, while the eighteen clusters account for 46.5% with 300 tracts.

## DISCUSSION

Circumstances surrounding every death are unique, so the evaluation of ecological factors related to suicide can be especially challenging. Prior literature has identified common risks that include limited access to care, socio-cultural factors, rurality and mental or behavioral health conditions. [3–8, 15–20] The characteristics of tracts identified through our final models or SaTScan clustering are consistent with earlier findings in the US, that suicide is associated with older age, non-Hispanic Caucasian population, and a somewhat more rural, non-metropolitan core geography. There was even evidence of an association between suicide and increased prevalence of depression and cigarette smoking.

Undetermined intent deaths are much less investigated and appear strongly associated with metropolitan cores, non-Caucasian population, younger age, area deprivation and limited access to care, for both the predicted response and SaTScan clusters identified. Though some colinearity exists between these features, particularly MUAs and ADI, many of model effects appear to affirm socially mediated disadvantages worsening rate of death. One exception seems to be limited access to care and suicide rate, but this finding controls for area deprivation in the same model. An inverse effect is at least consistent with previously reported nuance in interpreting ecological effects in suicide literature, especially for poverty, education and employment. [15, 17, 19, 20, 22] For example, one such study found a significant positive association between unemployment and suicide was inverted after including education and marital status for model effects. [17]

It also seems plausible that medical examiners have difficulty in assessing many of these deaths especially when no clinical or ecological information exists, beyond the scene of death. Social determinants, diagnoses, treatments or recent encounters might corroborate suspicions for intention of suicide. Leaving these cases as undetermined has the unfortunate effect of undercounting incidence for minority groups and further limiting stakeholders from recognizing communities most in need.

Our supplemental analysis of clusters by cause of death found consistency in regions identified by firearm and suicide deaths, and overdose/poisoning and undetermined deaths. This intersection of causes and manners is striking, since it is intuitively much easier for medical examiners to infer fatal intent for a firearm death than for an overdose. Therefore, another avenue for researchers to explore intentionality might be enhanced classification and documentation of causes of death.

These findings are reflective of only one state and a brief observation period. Counts for suicide death are limited, so we are unable to subdivide Census tracts further by age, sex, race or sub-intervals of time while maintaining a sufficient numerator for significance testing. This forces us to consider larger geographies for clustering in future research, such as the zip code tabulation area.

It is also unclear which parameters settings are best for the SaTScan technique and whether the same loadings are appropriate for both outcomes. We selected a large limit to tracts per cluster and found very different patterns for suicide and undetermined deaths. We have also experimented with flexible scanning statistics using different alpha settings and felt the results became much less intuitive for a ‘hot spotting’ interpretation. In the end, policy makers and care providers should be able to understand a region as having elevated risk or not, without having to question contiguity or shape.

Finally, we have demonstrated how certain regions, such as within Baltimore city, are found to have elevations in undetermined manner of death but not suicide, and even though the predictive models suggest similar associations between the two outcomes globally. These findings corroborate our own earlier work and support the view that there is a volume of suicide deaths being labeled as undetermined in manner. [5] This undercount is disproportionately affecting communities we may be able to identify using these techniques. Future work should focus on conducting retrospective psychological autopsies of these cases where intent seems likely, or by further stratification of undetermined deaths by age, sex or race. Temporal aspects of clustering and data quality must also be considered, to establish stability over time and in understanding historical effects, like the COVID-19 pandemic.

## CONCLUSION

We found cumulative suicide rate in the State of Maryland is greatest among more rural, non-minority Caucasian communities and undetermined deaths more associated with limited access to care, poverty, depression, alcohol misuse and non-Caucasian minority populations in core metropolitan areas of the same years. Medical examiners identify manner of death with a very limited ability to discern intent of the deceased. Care providers may be in a better position to evaluate plausible death by suicide but could benefit from the geographic context, especially from social determinants, access to care and disease prevalence. A detailed understanding of the ecological factors related to suicide incidence should inform policy makers and care providers in targeting mental health crises at the community level. Knowledge of where undetermined manner deaths are more common is valuable information for contextualizing risks further, especially where access to care is limited or suicide cases may be missed.

## Supplementary Material

This is a list of supplementary files associated with this preprint. Click to download.

- SUPPLEMENTALMATERIALS.docx

## Figures and Tables

**Table 1:** Model effects as Incidence Risk Ratio (IRR) for each outcome and model fit 1) M1: the base model consisting of area demographics, 2) M1 plus features for access to care (MUAs and RUCA), plus ADI, 3) M1 plus all features, including PLACES derived crude estimates of physical and mental health conditions. A pseudo R^2^ (pR^2^) statistic is provided in column headers for each fit, as an overall measure of model performance for null deviance explained.

Variable	Suicide			Undetermined		
	M1 (pR^2^ = 0.379)	M1 + ACCESS + ADI (pR^2^ = 0.417)	M1 + ACCESS + ADI+PLACES (pR^2^ = 0.450)	M1 (pR^2^ = 0.034)	M1 + ACCESS + ADI (pR^2^ = 0.436)	M1 + ACCESS + ADI+PLACES (pR^2^ = 0.605)
Population	1.000 (1.000:1.000)	1.000 (1.000:1.000)	1.000 (1.000:1.000)	1.000 (1.000:1.000)	1.000 (1.000:1.000)	1.000 (1.000:1.000)
% Male	1.001 (0.997:1.006)	1.003 (0.998:1.007)	0.999 (0.994:1.003)	0.982 (0.976:0.989)	0.993 (0.989:0.998)	0.992 (0.988:0.997)
Median age	1.003 (1.001:1.006)	1.012 (1.009:1.015)	1.014 (1.009:1.018)	0.980 (0.976:0.984)	1.013 (1.010:1.016)	1.021 (1.017:1.025)
% Minority race/ethnicity	0.990 (0.989:0.990)	0.988 (0.987:0.989)	0.992 (0.990:0.993)	1.000 (1.000:1.001)	0.988 (0.987:0.989)	1.003 (1.001:1.004)
MUA	-	0.930 (0.877:0.985)	0.840 (0.787:0.896)	-	1.770 (1.673:1.873)	1.137 (1.075:1.202)
National rank ADI	-	1.007 (1.006:1.008)	1.005 (1.004:1.007)	-	1.023 (1.022:1.024)	1.008 (1.006:1.009)
Metropolitan area, noncore	-	1.095 (1.047:1.144)	1.041 (0.994:1.092)	-	1.020 (0.962:1.081)	0.787 (0.745:0.830)
Micropolitan area	-	0.946 (0.853:1.050)	0.919 (0.827:1.022)	-	0.713 (0.627:0.812)	0.708 (0.633:0.793)
Small town, rural	-	0.817 (0.722:0.924)	0.810 (0.717:0.915)	-	0.371 (0.315:0.436)	0.409 (0.355:0.471)
Alcohol misuse crude prevalence	-	-	1.004 (0.991:1.017)	-	-	1.060 (1.046:1.074)
Cigarette smoking	-	-	1.087 (1.071:1.105)	-	-	1.086 (1.069:1.103)
Depression	-	-	1.009 (0.992:1.025)	-	-	1.134 (1.116:1.152)
Poor mental health	-	-	0.938 (0.919:0.957)	-	-	0.906 (0.888:0.923)
Obesity	-	-	1.002 (0.995:1.009)	-	-	1.017 (1.009:1.024)
Poor physical health	-	-	0.953 (0.929:0.979)	-	-	1.032 (1.008:1.057)
Reduced sleep	-	-	0.983 (0.970:0.996)	-	-	0.982 (0.969:0.996)

**Table 2:** Tract characteristics for regions identified as having elevated suicide incidence by 1) percentile binned model response and 2) clusters identified through descending order of SaTScan statistic. Clusters are identified as follows: a) C1, the primary cluster associated with the highest likelihood ratio, b) C2, the cluster associated with the second highest likelihood ratio, c) C3 + all remaining clusters identified as having a significant statistic where p < 0.01, and d) all regions not grouped in SaTScan clusters, and therefore not significantly elevated for suicide incidence.

Feature	Model response				SaTScan clusters			
	≤ 50th %tile	50th-74th %tile	75th-89th %tile	≥ 90th %tile	Not Clustered	C3+	C2	C1
Tracts	692 (50.0%)	346 (25.0%)	208 (15.0%)	139 (10.0%)	1,164 (84.0%)	131 (9.5%)	44 (3.2%)	50 (3.6%)
Cases	1,863 (38.4%)	1,283 (26.5%)	888 (18.3%)	815 (16.8%)	3,593 (74.1%)	728 (15.0%)	259 (5.3%)	269 (5.5%)
Population	2,364,910 (39.5%)	1,525,761 (25.5%)	1,088,439 (18.2%)	1,008,089 (16.8%)	4,966,808 (82.7%)	622,360 (10.4%)	205,986 (3.4%)	208,281 (3.5%)
Average population density (per km^2^)	6,744.3 (6,364.5)	3,537.3 (3,883.1)	2,580.4 (2,991.6)	1,941.5 (2,711.0)	5,522.2 (6,136.4)	539.4 (700.3)	4,483.2 (3,621.7)	1,936.2 (2,596.5)
Average median age	38.6 (7.2)	41.36 (6.4)	41.7 (6.0)	40.5 (6.3)	39.6 (7.1)	42.7 (6.2)	40.9 (4.5)	41.9 (5.2)
Average % male	47.8 (4.2)	48.8 (4.3)	48.9 (4.0)	49.0 (2.4)	48.2 (4.7)	49.6 (2.7)	48.6 (2.4)	50.8 (9.2)
Average % minority	61.6 (30.5)	31.1 (22.5)	23.5 (18.7)	20.7 (17.5)	49.8 (30.9)	15.1 (10.4)	16.1 (13.7)	15.0 (14.0)
Metro core	655 (94.7%)	258 (74.6%)	144 (69.2%)	93 (66.9%)	1,039 (89.3%)	31 (23.7%)	44 (100%)	40 (80%)
Metro non-core	24 (3.5%)	66 (19.1%)	51 (24.5%)	42 (30.2%)	96 (8.2%)	77 (58.8%)	0 (0%)	10 (20%)
Micropolitan	7 (1%)	15 (4.3%)	7 (3.4%)	2 (1.4%)	17 (1.5%)	14 (10.7%)	0 (0%)	0 (0%)
Small town, rural	6 (0.9%)	7 (2%)	6 (2.9%)	2 (1.4%)	12 (1%)	9 (6.9%)	0 (0%)	0 (0%)
MUA	196 (28.3%)	19 (5.5%)	11 (5.3%)	0 (0%)	226 (19.4%)	0 (0%)	1 (2.3%)	0 (0%)
Average national rank ADI	41.3 (30.5)	31.7 (25.2)	33.0 (24.2)	26.7 (20.1)	36.4 (28.6)	27.8 (19.2)	33.5 (26.6)	55.6 (23.3)
Average % alcohol misuse	14.9 (2.9)	15.4 (2.3)	15.8 (1.9)	16.8 (1.9)	15.1 (2.6)	16.9 (2.2)	16.7 (1.8)	14.6 (1.9)
Average % cigarette smoking	15.6 (6.1)	15.1 (4.8)	16.3 (4.7)	16.6 (3.4)	15.4 (5.6)	16.3 (3.2)	18.4 (4.3)	19.3 (3.8)
Average % depression	16.5 (3.3)	18.2 (2.3)	19.0 (2.2)	19.1 (1.7)	17.1 (3.0)	19.2 (1.3)	20.2 (1.8)	20.8 (2.0)
Average % poor mental health	13.9 (3.6)	13.3 (2.8)	13.8 (2.7)	13.6 (2.3)	13.6 (3.3)	13.7 (1.8)	14.4 (3.0)	15.8 (2.5)
Average % obesity	34.2 (7.7)	31.8 (6.0)	32.7 (5.1)	33.9 (4.2)	33.1 (7.1)	34.5 (3.8)	32.5 (3.0)	38.6 (3.6)
Average % poor physical health	11.3 (3.5)	11.0 (2.6)	11.5 (2.5)	10.9 (1.8)	11.0 (3.1)	11.4 (1.8)	11.9 (2.6)	14.1 (2.6)
Average % reduced sleep	40.8 (6.9)	35.9 (4.4)	35.9 (3.7)	36.2 (3.1)	38.8 (6.4)	35.4 (3.4)	35.8 (3.3)	38.0 (3.4)

**Table 3:** Tract characteristics for regions identified as having elevated undetermined death incidence by 1) percentile binned model response and 2) clusters identified through descending order of SaTScan statistic. Clusters are identified as follows: a) C1, the primary cluster associated with the highest likelihood ratio, b) C2, the cluster associated with the second highest likelihood ratio, c) C3 + all remaining clusters identified as having a significant statistic where p < 0.01, and d) all regions not grouped in SaTScan clusters, and therefore not significantly elevated for undetermined death incidence.

Feature	Model response				SaTScan clusters			
	≤ 50th %tile	50th-74th %tile	75th-89th %tile	≥ 90th %tile	Not Clustered	C3+	C2	C1
Tracts	692 (50.0%)	346 (25.0%)	208 (15.0%)	139 (10.0%)	1089	206	45	49
Cases	3,043 (28.6%)	2,698 (25.4%)	2,715 (25.5%)	2,178 (20.5%)	5,688 (53.5%)	3,147 (29.6%)	679 (6.4%)	1,120 (10.5%)
Population	2,778,392 (46.4%)	1,574,819 (26.3%)	996,246 (16.6%)	637,742 (10.7%)	4,979,259 (82.9%)	777,486 (13.0%)	109,885 (1.8%)	136,805 (2.3%)
Average population density (per km^2^)	4,133.2 (4,342.5)	4,209.3 (5,475.2)	5,509.2 (6,347.1)	8,885.3 (7,031.2)	3,816.3 (4,444.3)	6,078.3 (5,658.2)	17,536.2 (12,895.6)	12,150.4 (5,483.0)
Average median age	40.8 (7.2)	40.0 (6.8)	39.5 (6.2)	36.5 (5.8)	40.5 (6.9)	38.8 (6.6)	34.5 (5.6)	37.9 (6.1)
Average % male	48.5 (3.8)	48.7 (4.5)	48.1 (4.2)	46.9 (4.0)	48.6 (4.7)	47.9 (3.7)	48.3 (8.5)	47.1 (4.6)
Average % minority	47.68 (30.7)	32.9 (27.6)	39.6 (31.0)	61.2 (33.5)	42.1 (30.3)	38.0 (29.2)	70.0 (27.6)	92.2 (13.1)
Metro core	598 (86.4%)	243 (70.2%)	173 (83.2%)	136 (97.8%)	864 (79.3%)	196 (95.1%)	45 (100%)	49 (100%)
Metro non-core	67 (9.7%)	80 (23.1%)	33 (15.9%)	3 (2.2%)	173 (15.9%)	10 (4.9%)	0 (0%)	0 (0%)
Micropolitan	13 (1.9%)	16 (4.6%)	2 (1%)	0 (0%)	31 (2.8%)	0 (0%)	0 (0%)	0 (0%)
Small town, rural	14 (2%)	7 (2%)	0 (0%)	0 (0%)	21 (1.9%)	0 (0%)	0 (0%)	0 (0%)
MUA	56 (8.1%)	39 (11.3%)	52 (25%)	79 (56.8%)	80 (7.3%)	60 (29.1%)	39 (86.7%)	48 (98.0%)
Average national rank ADI	24.6 (21.5)	34.5 (23.5)	51.4 (25.4)	75.0 (24.9)	28.7 (23.1)	56.4 (25.3)	70.7 (29.8)	86.8 (13.1)
Average % alcohol misuse	14.7 (2.4)	16.4 (2.8)	16.0 (2.1)	14.9 (2.4)	15.2 (2.6)	16.0 (2.3)	16.8 (4.2)	13.5 (1.6)
Average % cigarette smoking	14.4 (3.4)	16.2 (3.0)	19.4 (3.3)	25.2 (5.2)	14.0 (3.9)	21.0 (4.3)	22.6 (7.6)	26.2 (4.9)
Average % depression	15.6 (2.5)	18.7 (2.0)	19.7 (1.5)	21.2 (2.0)	16.7 (2.8)	20.6 (1.8)	20.4 (1.3)	19.9 (1.8)
Average % poor mental health	12.1 (2.3)	13.9 (2.3)	15.4 (2.2)	18.7 (3.3)	12.8 (2.5)	16.5 (3.0)	17.7 (3.8)	19.0 (3.0)
Average % obesity	30.7 (6.7)	33.6 (5.2)	36.5 (4.3)	41.1 (5.4)	32.1 (6.4)	36.3 (4.8)	38.3 (8.5)	44.0 (3.8)
Average % poor physical health	9.8 (1.8)	11.2 (2.2)	12.8 (2.3)	16.2 (3.9)	10.4 (2.1)	13.5 (2.9)	14.2 (5.3)	17.5 (3.4)
Average % reduced sleep	37.2 (6.4)	37.2 (4.3)	40.0 (4.8)	44.8 (5.7)	37.2 (5.7)	40.3 (4.6)	45.3 (6.4)	49.1 (2.7)

## Data Availability

Data supporting this work is not publicly available nor permitted for sharing under the established data use agreements with multiple data providers. Some additional supplemental results and aggregated counts may be available upon request, and with the express permission of relevant data providers.

## Abbreviations

MSDW: Maryland Suicide Data Warehouse
SDH: Social Determinants of Health
CRISP: Chesapeake Regional Information System for our Patients
OCME: Office of the Chief Medical Examiner
ACS: American Community Survey
RUCA: Rural-Urban Commuting Area
AHRF: Area Health Resource File
HRSA: Health Resources and Services Administration
BRFSS: Behavioral Risk Factor Surveillance System
MUA: Medically Underserved Area
ADI: Area Deprivation Index
IRR: Incidence Rate Ratio
